# Phosphodiesterase 4B: Master Regulator of Brain Signaling

**DOI:** 10.3390/cells9051254

**Published:** 2020-05-19

**Authors:** Amy J. Tibbo, George S. Baillie

**Affiliations:** Institute of Cardiovascular and Medical Sciences, University of Glasgow, Glasgow G12 8QQ, UK; a.tibbo.1@research.gla.ac.uk

**Keywords:** phosphodiesterase, cyclic-AMP, rolipram, PDE4B, neuroinflammation

## Abstract

Phosphodiesterases (PDEs) are the only superfamily of enzymes that have the ability to break down cyclic nucleotides and, as such, they have a pivotal role in neurological disease and brain development. PDEs have a modular structure that allows targeting of individual isoforms to discrete brain locations and it is often the location of a PDE that shapes its cellular function. Many of the eleven different families of PDEs have been associated with specific diseases. However, we evaluate the evidence, which suggests the activity from a sub-family of the PDE4 family, namely PDE4B, underpins a range of important functions in the brain that positions the PDE4B enzymes as a therapeutic target for a diverse collection of indications, such as, schizophrenia, neuroinflammation, and cognitive function.

## 1. Introduction

**Figure 1:**
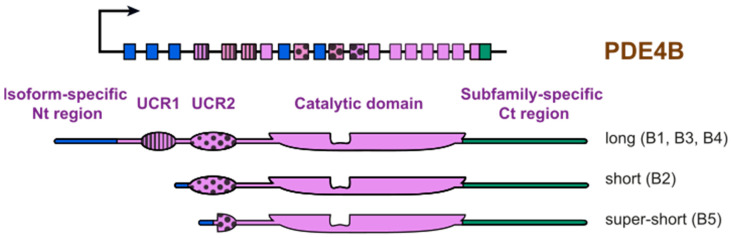
Scheme of PDE4B isoforms. The PDE4B subfamily generates five distinct isoforms. Each contains an isoform-specific N-terminal region, which is encoded for by specific exons denoted in blue. The family members are further specified through the presence of the upstream conserved regions 1 and 2 (UCR1 (stripes) and UCR2 (spots), respectively). All isoforms share an identical catalytic domain (purple) and C-terminal (Ct region) encoded by the green colored exon.

Cyclic nucleotides are ubiquitous signaling molecules that are recognized as archetypal second messengers. Since their discovery, there has been an unprecedented drive to understand signal transduction systems that utilize them and to characterize physiological systems under their control. It is well established that both cyclic adenosine monophosphate (cAMP) and cyclic guanosine monophosphate (cGMP) signaling systems underpin critical pathways necessary for brain development and function [1,2,3,4]. CAMP is generated by adenylyl cyclase (AC) following activation of G_s_-protein coupled receptors (GPCR) [5]. Recent appreciation of the discrete cellular positioning of these receptors and AC has supported the concept of compartmentalized cAMP signaling [6,7] that explains the ability of one second messenger to evoke a range of outcomes [8]. The spatial and temporal control of cAMP within cells is maintained via the action of a highly localized super family of enzymes called 3′5′-cyclic nucleotide phosphodiesterases (PDEs) [9,10,11]. PDEs are the only known enzymes capable of degrading cyclic nucleotides positioning them as highly important intracellular signaling regulators [12,13]. The distinct localization of PDEs results in hydrolysis of discrete pools of cAMP, controlling the duration of signal transduction in cellular nano-domains [14,15,16]. There are 11 families of PDEs (PDE1-11) that are the product of differential splicing of 21 genes. The cAMP specific PDE4 family is divided into four sub-families (A, B, C, and D; reviewed in [17]). Within PDE4B sub-family, five members exist (PDE4B1-5) consisting of long, short, and super-short isoforms (Figure 1).

Analysis of mRNA levels has identified that, along with PDE4D, PDE4B is the most abundant sub-family within in human, monkey, and rat brain [18]. Investigations of the roles of PDE4 in the brain have been by encouraged by the discovery that the selective-PDE4 inhibitor, known as rolipram (4-[3-(cytopentyloxy)-4 methoxyphenyl]-2-pyrrolidinone) (IC_50_ for PDE4B approximately 130 nM), promotes anti-depressant effects [19,20]. Rolipram possesses a 100-fold higher selectivity for PDE4 over the other subfamilies [21]. These studies now span a wide variety of neurological and central nervous system (CNS) disorders such as schizophrenia and traumatic brain injury [22,23,24,25]. Hence, investigations of the changes in PDE levels and activity in the brain during neurological development, aging, and disease are relevant to both basic mechanistic research and more translation studies [24,26,27,28,29,30,31]. This review discusses current opinions about the role of PDE4B in the brain and in brain and related diseases.

## 2. Structure and Activation of PDE4B Enzymes

Found on chromosome 1p31.3, the *PDE4B* gene gives rise to five protein isoforms in mammals, PDE4B1-5 [32]. Although individual isoforms contain highly conserved catalytic regions, they can be separated on a structural level by regulatory regions and unique N-terminal domains. Via the presence and size of the Upstream Conserved Region (UCR1) and UCR2 domains, isoforms can be categorized into long, short, super-short, or dead-short categories [33]. Within the PDE4B sub-family exist: three long isoforms, PDE4B1 (736 a.a), PDE4B3 (721 a.a), and PDE4B4 (659 a.a); a short isoform PDE4B2 (564 a.a); and a super-short isoform, PDE4B5 (484 a.a) (see Figure 1) [34,35,36]. Long forms contain both the UCR1 and UCR2 domains, short forms contain only the UCR2 domain, and super-short forms possess a truncated UCR2 region [33].

The UCR regions are responsible for modulating phosphorylation-dependent changes in enzymatic activity [33,37,38,39]. For example, UCR1 contains a conserved protein kinase A (PKA) site crucial for activation of all long isoforms. Long-form PDE4Bs also are known to contain a regulatory extracellular signal-regulated kinase (ERK) phosphorylation site at the end of the catalytic domain [38]. In contrast to the long PDE4B isoforms, which are inhibited by ERK phosphorylation, short-form PDE4B2 is activated by the same modification [38]. PDE4B’s ability to bind to the kinase is a function of an ERK specificity motif (Phe693, Glu694, Phe695; FQF motif) located on an α-helix around 5–30 amino acids downstream of the targeted serine [39]. The catalytic domain of PDE4B enzymes also contain a more general kinase interacting motif (KIM) on a β-hairpin loop [39]. These two motifs allow for the docking and subsequent phosphorylation by ERK2.

It has been well established that the formation of dimeric long-form PDE4 complexes define the properties of the enzyme [40,41,42]. Dimerization is mediated by the UCR1 domain and, given the sequence conservation, PDE4s can form both homo- and heterodimers. The dimerization of PDE4B has been shown to be critical for the regulation of the enzyme. As mentioned, PDE4B is activated by PKA-dependent phosphorylation. The structural basis of the phospho-dependent activation and inhibition of PDE4B enzymes has been suggested by crystallographic studies, which identified the mechanism by which the UCR1 domain of one monomeric subunit of the long isoform PDE4B1 crosses over and allows for the regulation of the catalytic activity of the other monomeric subunit [37].

## 3. Role of PDE4 Isoforms in Cognitive Function and Memory

The prevalence of age-related diseases of the brain has grown exponentially in accordance with an increased life expectancy [43]. Cognitive dysfunction is a characteristic feature of age-related memory decline and although research regarding the loss of cognition and memory impairment has been considerable, the need for successful targeted treatment remains high. Memory formation is underpinned by gene expression controlled by the cAMP response element binding protein (CREB), a transcription factor activated by PKA phosphorylation [24]. The conversion to long-term memory depends on both transcription and translation [44]. In mice behavioral studies, the deficiency of CREB as well as the increase of CREB-binding protein (CBP) identified a key role for the transcription factor in the facilitating of long-term memory storage [45]. Long-term potentiation (LTP) is transcription-dependent synaptic plasticity, which has been extensively utilized as an investigatory model from long-term memory [46]. There are temporally different LTP stages that are broken down into three categories: short-term potentiation (STP), early-LTP and late-LTP [46]. These stages are dependent upon a transient increase in cAMP levels, PKA activation and CREB phosphorylation [47]. As PDEs modulate cAMP levels within cells, there has been increased interest PDE inhibition to improve cognitive function.

In the brain, PDE4B has been detected in the amygdala, thalamus, hypothalamus, white matter tracts, striatum, and, importantly, the hippocampus [18,43,48,49,50]. Hippocampal neurons possess unique properties that allow them to promote memory formation. Hippocampal LTP is the best-characterized cellular model for investigating learning and memory formation [51,52]. The first association of PDE4B with learning and memory was discovered in an investigation of long-term potentiation (LTP) in hippocampal neurons. Ahmed and Frey [51] identified PDE4B3 as the first cAMP-specific phosphodiesterase to be associated with the control of LTP stages in rat hippocampal slices. There is documented evidence that late-LTP is dependent upon a transient increase in cAMP levels, which results in the phosphorylation of transcription factors such as CREB. Two studies have independently shown that a mouse double AC knockout mutant failed to transition to late-LTP supporting the notion that cAMP modulation in neurons is important for late-LTP [51,53,54]. Interestingly, both the PDE4B3 transcript and PDE4B3 protein fluctuated during transition through the different stages of LTP in area cornu ammonis (CA1, the first region of the hippocampus) leading to the stage dependent transient modulation of cAMP [51,55]. Delayed cAMP elevation could be involved in the PKA dependent process of covalent modifications and as such creating a functional state of “plasticity-related proteins” (PRPs) [56]. PRPs may be directly involved in maintaining LTP processes. This mechanism remains unclear but has been named the ‘synaptic tagging and capture hypothesis’. This proposes that a strong synaptic pathway leads to two pathways: local tag setting and the synthesis of PRPs. The PRPs are captured by tagged synapses allowing the sustenance of LTP maintaining it up to 8 h [57]. The delay in the elevation of cAMP levels may be crucial to the function of such tag molecules and PRPs driving LTP into its late stage. PDE4B may be characterized as a secondary PRP due to modulation of cAMP levels that promote the process through initial to late stage. As such PDE4B would function to modulate the synaptic plasticity stimuli within the cell and allow for the change in reaction later in the memory forming process [57].

In humans and rodents, the tendency to explore their environment is a commonly used characteristic to evaluate generalized cognitive abilities; however, an increase in exploration does not directly correlate with improved cognitive function [58,59]. Through the reduction of aversive stimuli, such as loud noises and bright light, leads to a shift in the motivation, which underlies environmental exploration therefore facilitating learning [60]. PDE4B^Y358C^mutant mice, containing a catalytic domain mutation reducing its cAMP hydrolytic ability in a C57BL/6J mouse background, displayed decreased fear responses and increased exploration accompanied with cognitive enhancement during non-aversive tests [61]. During fear conditioning in the lateral amygdala, where PDE4B is highly expressed, inputs are formed through associations between the conditioned and unconditioned stimulus [62]. The decrease in fear responses in PDE4B^Y358C^ mice is consistent with the upregulation of β-arrestin and disrupted in schizophrenia 1 protein (DISC1) and decrease of the β-arrestin-PDE4B interaction, which is crucial to the formation of associative fear [61,63]. The PDE4B^Y358C^ mutant mice fear conditioning phenotype was replicated using a subchronic dose of rolipram (1 mg/kg twice daily), which inhibits all PDE4 activity and does not interfere with the interaction of the complex [24]. The same phenotype was replicated when rolipram was administered 24 h after fear conditioning, pointing to a role for PDE4B in the late signaling processes required for long-term memory persistence [24]. The impaired cAMP signaling in the hippocampus due to PDE4B may impair the very late-phase consolidation via poor coordination of the late-phase protein transcription required for long-term memory persistence due to a dysregulation in CREB activation. Given that the fear conditioning phenotype of PDE4B^Y358C^ was replicated by rolipram treatment, which does not interfere with the interaction with DISC1, it was suggested that reduced contextual freezing is a result of PDE4B dysfunction rather than the disruption of the DICS1/PDE4B complex [24].

Although the information outlined above suggests that PDE4B activity is critical to the formation of memory, behavioral animal studies have provided contradictory evidence. In step down passive avoidance and the Morris water test, two tests that measure hippocampal based memory, PDE4B^-/-^ mice showed no significant change in either short- or long-term memory, in fear conditioning or in acquisition [61,64]. Electrophysiology readings examining excitatory post-synaptic potentials from PDE4B^-/-^ mice hippocampal slice samples did however identify an increase in the proliferation of neuronal cells in the hippocampal dentate gyrus along with enhanced basal postsynaptic responses and long-term depression [61]. It is possible that the loss of protein in other regions of the hippocampus and in the brain as a whole caused by the knockout of PDE4B could affect the neuronal circuitry within the CA1 region in turn leading to synaptic plasticity. Potentially, the treatment with specific PDE4B inhibitors could provide therapeutic relief for patients with persistent fear memories and post-traumatic stress disorders through the alteration of PDE4B activity.

## 4. The DISC1-PDE4B Signalosome in Schizophrenia

The PDE4 family is the most intensively examined family of PDEs in the context of neurological disorders. Over the years it has been firmly established that PDE4 family members are widely and differentially expressed throughout the brain [35]. In particular, all of the five PDE4B isoforms are found within the brain and this is the predominant sub-family in the amygdala, hypothalamus, and striatum [18,36,48]. Both, the amygdala and the hypothalamus, are key regions in the control of anxiety and responses to stress [65] and it is not surprising that PDE4B is of particular importance in these areas. The role of PDE4B in the field of molecular psychiatry was brought to prominence because of its link with the DISC1 gene [23,66].

The DISC1 gene is a potential susceptibility factor for psychiatric illnesses, which has been shown to be disrupted by a balanced chromosomal translocation (t(1;11)) in a number of cases of schizophrenia [23,67,68]. The t(1;11) translocation results in the production of an abnormal product due to the fusion of the DISC1 gene on chromosome 1 with a disrupted gene on chromosome 11 [69]. From this translocation, the expression of the three possible chimeric transcripts, designated; CP1, CP60, and CP69, resulted in a profoundly altered, deleterious function [69]. Given this information, it has been reported that the reduction in DISC1 function and expression is consistent with a haploinsufficient disease.

DISC1 dysregulation is not the sole contributing factor to Schizophrenia susceptibility. Initial genetic evidence for PDE4B‘s role in Schizophrenia was discovered in two cousins, both harboring the same translocation disrupting PDE4B and been diagnosed with the psychiatric disorder [23]. In patients with the balanced (1;16) translocation there was an accompanied ~50% reduction in the protein expression of the long PDE4B1 isoform [23]. Reduction in the levels of PDE4B in the postmortem brains of schizophrenia patients prompted the question whether PDE4B may be a common component in a variety of neurological disorders. This notion was supported by recent work suggesting that novel single nucleotide polymorphisms (SNPs) in PDE4B are associated with an increase incidence of schizophrenia within the general population [70].

To determine the molecular function of PDE4B in schizophrenia signaling processes, research groups have investigated the DISC1–PDE4B complex. From transfected HEK293 cell lysates, successful co-immunoprecipitation of DISC1 and PDE4B1, PDE4B3 and the short form PDE4B2 was achieved, suggesting that a common DISC1 binding site is present in these three isoforms [23]. These findings were confirmed with endogenously expressed proteins from human neuroblastoma-derived cell lines SH-SY5Y and LAN5 as well as in primary rat hippocampal cells. The DISC1 interactome was investigated using yeast-two hybrid (Y2H) technology, which also identified PDE4B as an interacting protein [71]. Truncation of PDE4B1 by removal of the UCR2 domain resulted in a loss of DISC1 interaction. This suggested that the specific interaction domain was in the UCR2 domain of PDE4 isoforms, which was confirmed using the UCR2 domain alone to co-immunoprecipitate DISC1 [23]. Peptide array analysis using full length long-forms PDE4D5 and PDE4B1, overlaid with DISC1, identified two common sites in the UCR2 domain [66]. However, PDE4B1 arrays overlaid with a [^35^S]methionine radiolabelled-DISC1 also highlighted a specific binding site for DISC1 in the catalytic domain of PDE4B1 [66]. Cell penetrating peptides containing the sequence of the two common sites were unable to disrupt the interaction providing further support that PDE4B14B1 utilizes isoform specific binding regions. This concept was further supported utilizing point mutations in the PDE4B1 specific binding sites, which led to reduced DISC1 binding to PDE4B in both human and mouse variants [66]. Reciprocal arrays identified two common sites on DISC1 crucial to the binding of both PDE4B and PDE4D subfamilies; however, a further three sites were specific to PDE4B1 only. These residues were confirmed to be crucial to the interaction through the loss of binding between PDE4B1 and an N-terminally truncated DISC1 [66]. Intriguingly, the super-short isoform PDE4B5 was also found to interact with DISC1 meaning that the interaction motif must lie in the residual part of the truncated UCR2 or in an unknown site within the catalytic domain [34]. The presence of unique binding sites for PDE4B1 supported the notion that it has higher affinity for DISC1 than PDE4D5. 

It is noteworthy that the interaction of PDE4B and DISC1 is dynamic and is thought to be regulated by cellular levels of cAMP via a PKA mediated process. Treatment with forskolin (IC_50_ 41 nM) and the non-specific PDE inhibitor 3-isobutyl-1-methylxanthine (IBMX; IC_50_ values of 6.5 ± 1.2, 26.3 ± 3.9 and 31.7 ± 5.3 μM for PDE3, 4 and 5 respectively) led to a marked decrease in amount of DISC1 precipitated with PDE4B [23]. These results suggested a potential model for the interaction, which involved phosphorylation of PDE4B by PKA that resulted in the dissociation from DISC1, in turn, allowing an activated PDE4B long-form pool to reduce local cAMP levels [23]. However, this was refuted by Murdoch et al. [66], who reported no changes in the interaction between DISC1 and PDE4B under differing cAMP concentrations.

What is the relevance of the interaction between DISC1 and PDE4B for signal transduction within the brain? A complex has been identified that contains Nuclear Distribution Factor-E-like (NDEL1) and its orthologue NDE1, Lissencephaly (LIS1), DISC1, and PDE4B. This complex is considered to have a crucial role in neuronal migration due to its localization at the centrosome [29,72,73]. The centrosome is in control of neuronal cell polarity and symmetry, the regulation of the neural progenitor pool and, crucially, the production of new neurons [74]. Additionally, the DISC1 and NDEL1 complex is responsible for the recognition of signals setting the migration pattern limits of neurons in adult brains [75]. Phosphorylation of NDEL1/NDE1 by PKA is known to modulate its protein-protein interactions as well as its subcellular localization [76,77,78,79]. The PDE4B/DISC1 interaction influences the PKA dependent phosphorylation of NDE1/NDEL1 at threonine-131/2 (T131/2), which can alter its interaction with LIS1 [76]. Phosphorylation at this site is proposed to modulate the production and positioning of neurons, neurite outgrowth, and LIS1-dependant synaptic function [29,76,80,81]. Reductions in PDE4B, like that seen with translocation mutations, would result in increased cAMP levels and subsequent activation of PKA activity. Given the discussed role of PKA phosphorylation in controlling NDE1/NDEL1s interactions, targeting PDE4B provides an interesting therapeutic strategy in this setting. Altered protein interactions within the multiprotein complex could drastically change neuron-signaling leading to progression of psychiatric disorders. Collectively, these data indicate that dysregulation of the PDE4B/DISC1interaction with NDE1/NDEL1/LIS1 complex induces the psychiatric disorder phenotype. This multiprotein complex identifies a new therapeutic avenue for numerous psychiatric disorders including schizophrenia. 

Currently, pan-PDE4 pharmacological inhibitors, such as rolipram, successfully generate neuroprotective and neurogenerative benefit however, an investigation of knock-out PDE4 mice has shown that individual PDE subtypes play a distinct and non-overlapping role [13]. Behavioral studies comparing PDE4B^-/-^ male mice to their PDE4B^+/+^ littermates revealed a moderate anxiogenic-like behavior measured by a decrease in immobility during forced swim test, reduced head-dipping in the hole board test as well as decrease exploration and rears in the open-field test [64]. However, the PDE4B^-/-^ mice performed as wild type (WT) mice in both passive avoidance tests and in fear conditioning, as well as showing unaltered reactions in a shock test [61,64,82]. Moreover, these mice also show a lack of inhibitory effects on conditioned avoidance responses when treated with Rolipram [83]. Specific inhibition of the catalytic domain of PDE4B using a PDE4B^Y358C^ mutant mouse resulted in an increased phosphorylation of CREB, decreased binding to DISC1 accompanied by increased DISC1 and β-arrestin expression in both the hippocampus and amygdala. These data points to a possible mechanism for compensatory changes designed to compensate for lack of PDE4B activity [24]. PDE4B^Y358C^ mice also displayed decreased anxiety, increased cognitive enhancement, enhanced neurogenesis and impaired depotentiation [24]. Interestingly, these mice displayed decreased contextual fear memory at 7 days post fear conditioning, which was also “phenocopied” using pharmacological inhibition of PDE4 with rolipram [24]. In terms of treatment, chronic use of antidepressants including serotonin reuptake inhibitors, such as citalopram and paroxetine, led to an upregulation in both PDE4B and PDE4A in the cerebral cortex [84]. In addition, chronic cocaine administration has been shown to downregulate the levels of PDE4B within the nucleus accumbens, a dopamine target area, leading to a profound inhibition of the reward actions of psychostimulants [84]. Therefore, modulation of the levels of PDE4B through specific antidepressant use may influence both motivation and reward.

The research outlined above highlights the utility of strategies designed to inhibit specific isoforms or sub-families of PDE4. Targeting PDE4B activity or PDE4B-containing signalosomes may provide a novel route for the development of novel compounds that treat CNS disease without causing the side effects produced by pan-PDE4 inhibition, such as emesis and nausea [85].

## 5. PDE4B in Neuroinflammation

**Figure 2:**
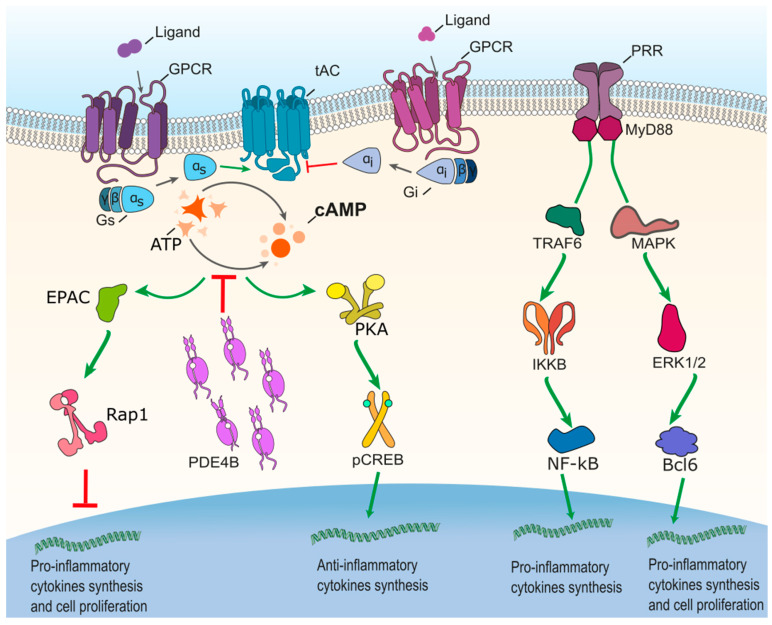
Schematic of inflammatory signaling pathways, PDE4B is involved in the production of inflammatory and anti-inflammatory cytokines through its action in degrading cAMP. PKA and Epac are activated by high cellular cAMP resulting in the phosphorylation of cAMP-responsive element binding protein (CREB) and activating transcription factor 1 (ATF1) leading to the generation of anti-inflammatory cytokines. Transcriptional activity of NF-κB can be regulated by PKA through modulation of its interaction with CREB. Phosphorylation of PKA can inhibit the synthesis and activity of B-cell lymphoma 6 protein (Bcl-6) and NF-κB–mediated proinflammatory cytokines. In addition, the activation of Epac leads to small GTPases (Rap1) blocking the release of pro-inflammatory cytokines. These pathways show PDE4 to be a viable target for reducing inflammation.

It is widely known that the PDE4 subfamily is heavily involved in the regulation of inflammatory cell activation [86,87,88]. Toll-like receptors (TLRs) within cells initiate responses to numerous pathogen-associated molecular patterns and host derived molecules [89,90]. The activation of TLRs initiates a downstream signaling cascade leading to the upregulation of a wide variety of target genes including chemokines, cytokines, and other inflammatory molecules [89,91,92]. Lipopolysaccharides (LPS) stimulate TLR4, which in turn drives activation of both Myeloid differentiation primary response 88 (MyD88) and Toll/interleukin-1 receptor-domain-containing adapter-inducing interferon-β (TRIF)-dependent pathway (Figure 2). Consequentially, there is the activation of numerous downstream pathways such as the nuclear factor κB (NF-κB), extracellular-signal regulated kinase 1/2 (ERK1/2) pathways, as well as the interferon regulatory factor 3 (IRF3) pathway, a transcriptional factor involved in expression of inflammatory cytokines (Figure 2) [90]. The stimulation of TLR4 by LPS and production of other regulators including cAMP elevators drives the expression of the interleukin-1 receptor antagonist (IL-1Ra) in both monocytes and macrophages. IL-1Ra is concomitantly produced in response to inflammatory stimuli and functions to downregulate pro-inflammatory cytokines such as tumor necrosis factor α (TNF-α) [93]. The complexity of this pathway driving proinflammatory-signaling means that dysregulation at any of the key regulators can lead to a prolonged inflammatory response, which if left unchecked, can create detrimental effects.

As mentioned above, cAMP can regulate endogenous inflammatory response mechanisms, with increasing cAMP concentrations promoting anti-inflammatory effects [94]. For example, augmented cAMP signaling is responsible for the upregulation of numerous inflammatory markers including Arginase 1 (Arg1) [95]. Specific inhibition of the cAMP-specific PDE4 significantly alters the functions of specialized inflammatory cells [96,97,98]. Wang and colleagues [86] identified PDE4B2 to be the most dominant isoform within human neutrophils, leukocytes, astrocytes and monocytes representing 95–100% of total cellular PDE4. PDE4B2 has been identified to control and regulate neutrophil accumulation as well as, importantly, TNF production [99,100]. In the context of inflammation, PDE4B2 is of particular importance because of its control over various inflammatory stimuli including induction by LPS and inhibition by Interleukin (IL) 10 4 [86,87,101]. Furthermore, increased expression in PDE4B resulted in a pro-inflammatory phenotype in neutrophils, macrophages, and microglia. This concept has been further strengthened following the discovery of increased PDE4B2 expression in experimental models of neuroinflammation diseases [87,101]. For example, PDE4B2 was found to exhibit elevated expression within infiltrating T cells, macrophages, and microglia following inflammatory induction of experimental autoimmune encephalomyelitis (EAE) in Lewis rats [31].

Microglia and macrophages are known to express multiple isoforms of PDE sub-families PDE4A, B, C, and D; however, PDE4B was identified to be the primary subfamily involved in the LPS response [87,99]. This was confirmed in PDE4B^-/-^ mice that exhibited a retarded TNFα response to LPS in both peripheral blood leukocytes as well as in peritoneal macrophages [99,102]. LPS specifically upregulates PDE4B expression and the resulting TNFα reduction is dependent on the cAMP/PKA pathway. No other cAMP-specific PDE was able to rescue this phenotype and in LPS-induced shock it was only PDE4B^-/-^ animals that were protected [99,102]. There is a lack of evidence surrounding genetic ablation of PDE4B in microglia; however, the general pharmacological inhibition of PDE4 with rolipram after TLR/LPS stimulation resulted in the reduction of proinflammatory cytokines, including tissue factor-1 (TF1) and TNFα, genes that are positively regulated by nuclear factor kappa B (NF-κB), as well as proinflammatory mediators, such as inducible nitric oxide synthase (iNOS) and cyclooxygenase-2 (COX-2) [103]. In fact, PDE4B^-/-^ mice showed a >50% reduction in both the levels of TNF-α mRNA and protein in macrophages, which was not seen with either PDE4A^-/-^ or PDE4D^-/-^ mice [99]. It has been established that the release of proinflammatory cytokines, TNFα, and IL-1β lead to a dramatic reduction in microglial cellular cAMP levels in conjunction with a marked increase in PDE4 activity and expression levels [87]. This finding was accompanied by the demonstration, using a cyclic AMP-Glo™ Assay (Promega), that PDE4 inhibition via rolipram or RNAi knockdown of PDE4B2 in microglia prevented the reduction of cAMP after TNF-α activation [87]. In addition, Alzheimer’s disease (AD) model mice, which are deficient in PDE4B, show decreased TNFα expression in response to inflammatory stimuli. Furthermore, Ghosh and colleagues [103] were able to reverse the cAMP reduction created by TNFα by inhibiting PDE4 activity with rolipram. 

Interestingly, after TNFα stimulation there was an increase in the levels of ERK1/2 activity ERK phosphorylation [38], and subsequently, an increased activation of PDE4B2, and other short isoforms, greatly reducing the cAMP levels thereby reducing inflammatory signals [87].

As described, elevation of intracellular cAMP downregulates a range of multifaceted immune cell functions including the expression of TNFα, interferon-γ (IFN-γ) and numerous ILs [104,105,106,107,108]. There is increasing evidence to support the hypothesis that an immune challenge by LPS creates depressive-like behavior, possibly related to the upregulated PDE4 activity that is associated with a neuroinflammatory response. Traumatic Brain Injury (TBI) is a serious clinical problem that can leave patients with high levels of cognitive impairment and lasting TBI-related deficiencies reducing both life quality and expectancy [25,109,110,111]. During an acute inflammatory response, cAMP levels are significantly repressed post-injury and remain so for several days [30,112]. Such a reduction in cAMP signaling severely attenuates CREB activation, which is a master regulator of pathways critical to memory and learning as well as an acute up-regulator of PDE4B2. Targeting TBI-directed inflammation has been identified as a promising strategy for reducing histopathological damage and cognitive deficits. As mentioned, PDE4B2 has been implicated in both the activation and regulation of inflammatory cells including microglia and neutrophils [87,101]. Within 24 h of a TBI, there is an immediate and extensive infiltration of the brain by neutrophils leading to pathophysiological symptoms, including neuroinflammation, [113] as well as elevated levels of PDE4B2 in both the injured cortex and the hippocampus [25]. Interestingly in models of inflammatory lung disease, PDE4B^-/-^ and PDE4D^-/-^ neutrophils also showed markedly decreased chemotaxis in response to chemokine (C-X-C motif) ligand 1 (CXCL1 or KC) and levels of macrophage inflammatory protein-2 (MIP-2) [100]. This effect was comparable to the PDE4 inhibition by rolipram, but was not additive. Importantly, this data underpinned the idea that both PDE4B and PDE4D share a complementary, non-redundant role in the control of neutrophil function [100], which provides precedent for the mechanism in the brain. 

More evidence of PDE4B involvement was uncovered when PDE4B^-/-^ mutant mice^-^ and mice treated with A33 (2- (4-phenyl)acetic acid, a selective PDE4B inhibitor with an IC_50_ = 27 nM [30,114,115]) showed a reduction in the levels of neutrophil accumulation after 24 h, but not 3 h after treatment. This suggests that the initial neutrophil response is not driven by PDE4B, but peak accumulation may be [25]. A33 selectivity of PDE4B is derived from a single amino polymorphism in the C-terminus, which inhibits its activity by keeping it locked in the inactive conformation. Following the inhibition of PDE4B by A33, cAMP levels were not reduced, leading to an increase in CREB phosphorylation and subsequent activation of anti-apoptotic genes, such as B cell lymphoma 2 [116]. Additionally, it is suggested that PDE4B2 inhibition decreases Integrin beta chain-2 (CD18) expression within the circulating neutrophils. CD18 expression is a crucial mediator for neutrophil infiltration [117,118]. Therefore, CD18 reduction in circulating neutrophils leads to the reduction in infiltrating neutrophils in the brain [100]. Such data suggest specifically targeting PDE4B is a valid therapeutic target for reducing the peak accumulation of neutrophil post TBI. As such, further investigations of TBI in model systems of the central nervous system (CNS) have already identified the use of PDE4 inhibitors, such as rolipram, and the elevation of cAMP levels, to be an effective strategy for reducing inflammation and promoting recovery [119,120]. Pre-injury treatment with rolipram has clearly been shown to rescue the cAMP signaling deficits in conjunction with reduced inflammation after TBI as well as reduced neuronal loss in the cortex and l CA3 region, the hippocampal region that receives inputs from the entorhinal cortex [22]. Although administration of rolipram post-TBI confirmed these concepts, exacerbation of the cortical contusion volume and atrophy was observed, resulting in prolonged brain injury [112,121]. Pan-PDE4 inhibitors have been used to reduce TNF levels and increase the accumulation of neutrophils in mouse models of CNS-related inflammatory disorders including TBI [122]. The issue with the general inhibition of PDE4 is that the benefits of acute treatment are overshadowed by vascular perturbations, shown by increased hemorrhage after TBI [22,25]. As PDE4B plays a crucial role in the modulation of inflammation and the subsequent TBI pathophysiology, adult male Sprague Dawley rats were induced with a moderate parasagittal fluid-percussion brain injury before being treated with either A33 or vehicle control [25]. Treatment after injury resulted in a pronounced reduction in microglial activation markers and neutrophil infiltration at 3 h and 24 h post injury. Specificity for PDE4B in this regard is under continuing investigation. Goto and colleagues [123] created a novel and potent *N*-propylacetamide derivative, known as compound 31b, for PDE4B (IC_50_ = 7.5 nM) and TNFα (IC_50_ = 9.8 nM) inhibition in mouse splenocytes. Although this compound has not been investigated in the brain, it can reduce anti-inflammatory activity in lung inflammation mouse models induced by LPS, which may be translated in the brain [123].

It is evident that PDE4B has a major role in neuroinflammation caused by injury, and it is also clear that the enzyme plays a critical role in the alcohol priming effect, leading to exacerbated responses of immune cells to LPS [124,125,126,127,128]. Work performed by McClain and colleagues [125] identified that there was a decrease in cellular cAMP levels, which had a pathological role in ethanol priming, which lead to an increase in the LPS-inducible TNFα expression. This is in part due to the knowledge that alcohol-induced, gut-mediated peripheral endotoxemia has a significant role in neuroinflammation and glial cell activation. Alcohol fed C57B1/6 wild type (WT) mice displayed both induced peripheral endotoxin and increased PDE4B expression in the brain [129,130]. This was accompanied by activation of both microglia and astrocytes, as well as a significant increase in levels of inflammatory cytokines and markers, including TNFα, as previously described, Monocyte chemoattractant protein-1, IL-17, and COX-2 [127,129,130,131]. Microglial cells activated by alcohol displayed increased levels of only the PDE4B sub-family [129], and this was validated by experiments where a single intraperitoneal administration of endotoxin significantly upregulated PDE4B expression in glial cells, as well as distinct CNS expression of numerous inflammatory mediators [49,50]. Interestingly, alcohol feeding in mice results in increased serum endotoxin levels with elevated cluster of differentiation 14 (CD14), causing gut dysfunction accompanied by a movement of microbial products, leading to a systemic innate immune response [129]. Accompanying the increase in endotoxin production was the activation of alcohol-induced inflammatory changes in the brain, including the activation of glial cells [129]. Furthermore, there was both an increase in TLR4 and PDE4B expression in both microglia and total brain homogenate. Another noteworthy point is that rolipram was found to significantly reduce alcohol intake and alcohol preference in mice [130,132]. Functional implications of *PDE4B* expression in alcohol-affected brains are related to the decrease in total cAMP levels that triggers loss of immune homeostasis, reductions in pro-inflammatory mediator release, and a diminution in the release of anti-inflammatory cytokines, such as IL-10 [133]. The pivotal role of PDE4B in alcohol promotion of inflammation has also been strongly supported by work in PDE4B^-/-^ mice that do not displaying the symptomatic decrease in cAMP that usually accompanies alcohol ingestion [129]. In conjunction with this, the inhibition of PDE4B activity by PDE4 inhibitors—rolipram (1 and 5 mg/kg) and piclamilast (IC_50_: 2 nM; 1 mg/kg)—led to a marked reduction in the production of inflammatory cytokines as well as in the activation of glial cells [133].

## 6. Conclusions

We have reviewed the role of PDE4B in brain development, brain function, and the in the pathophysiology of numerous brain-related diseases. Our take-home message is that the regulation, localization, and expression levels of PDE4B isoforms are crucial to the maintenance of healthy brain function. We feel it is apparent that a more targeted approach to treatment of diseases of the brain mentioned here could be achieved by pharmacological interventions that specifically attenuate activity of PDE4B isoforms or the discretely localized signaling complexes that contain this PDE4 sub-family. Unfortunately, the similarity of the structure of the catalytic/regulatory domains between PDE4B isoforms means that this level of selectivity has not been possible for small molecule inhibitors to date, and peptide disruptors that can specifically dislodge single PDE4B forms are unlikely to be able to cross the blood–brain barrier unless used in conjunction with novel transporter technology, such as nanoligand technology or BBB shuttle peptides [134,135,136]. We look forward to further exciting developments that signify new roles for the PDE4B sub-family in the brain that may offer tractable, mechanism-based therapeutic avenues.
